# Supplementary material for: A tuberculin skin test survey among healthcare workers in two public tertiary care hospitals in Bangladesh
Source: PLoS One. 2020 Dec 17;15(12):e0243951. doi: 10.1371/journal.pone.0243951 (PMC7745963; doi:10.1371/journal.pone.0243951)
Supplement: S1 File — (PDF) [file pone.0243951.s002.pdf]

TST Exposure questionnaire

ID No: □□□□□□□□□
**Name of facilities (হাসপাতালের ধরন):** ....   **Name of Ward (ওয়ার্ডের নাম)**   **Date: (**তারিখ)......../........../..........

1. **Age (বয়স)**: ………………………………………………….
2. DOB (Day/Month/ Year) [জন্ম তারিখ (দিন/মাস/বছর)]: ..../........../............../
3. Sex(লিঙ্গ): □ 1 = Male (পুরুষ) □ 2 = Female (মহিলা) □ 3=Transgender (ট্রান্সজেন্ডার)
4. History of BCG vaccination (by inspecting BCG scar)[বিসিজি টিকার ইতিহাস (বিসিজি টিকার দাগ দেখে)]

   1=Yes (হ্যাঁ)

   2=No (না)

   3= Do not know (জানিনা)

5. Address with location (স্থানের উল্লেখসহ ঠিকানা):

   Village/ Mahalla (গ্রাম/মহল্লা)______________________

   Union/ Ward (ইউনিয়ন/ওয়ার্ড) _______________________

   Upazilla/Municipality(উপজেলা/পৌরসভা):________________

   District (জেলা): _______________________________

   Locations: (অবস্থান):

6. . Phone/mobile number (প্রাইমারি ফোন নম্বর): 1.□□□□□□□□ □□□

6a.1) Who use this number? (এই ফোন নম্বরটি কে ব্যবহার করে?):

□ i. Self (নিজে) □ ii. Others (Please specify)[অন্য কেউ (নির্দিষ্ট করে লিখুন)]......................................

6b) Secondary number (সেকেন্ডারি ফোন নম্বর)..

6b.1) Who use this number? (এই ফোন নম্বরটি কে ব্যবহার করে?):

□ i. Self (নিজে) □ ii. Others (Please specify)[অন্য কেউ (নির্দিষ্ট করে লিখুন)] ....

7. .Level of education (Last completed class or degree) [শিক্ষাগত যোগ্যতা (সর্বশেষ সম্পন্নকৃত ডিগ্রী)]

   1=No schooling (নিরক্ষর)

2= primary (1-4 class) [প্রাথমিক অসমাপ্ত (১ম- ৪র্থ শ্রেণী)]

3=Finished primary (প্রাথমিক সমাপ্ত)

4=Some secondary (6-9 class) [মাধ্যমিক অসমাপ্ত (৬ষ্ঠ-৯ম শ্রেণী)]

5=Finished secondary (SSC) [মাধ্যমিক সমাপ্ত (এসএসসি)]

6= Finished higher secondary(HSC) [উচ্চমাধ্যমিক সমাপ্ত (এইচএসসি)]

7= Graduation (স্নাতক)

8=Masters (স্নাতোকোত্তর)

8. Monthly income of your house hold in BDT [পরিবারের মাসিক আয়(টাকা)]

1=0-10,000 (০-১০,০০০ টাকা)

2=10,001-20,000 (১০,০০১-২০,০০০ টাকা)

3=20,001-30,000 (২০০০১-৩০০০০ টাকা)

4=30,001-40,000 (৩০০০১-৪০০০০ টাকা)

5=40,001and above (৪০০০০ টাকার বেশি)

9. Type of profession/designation (পেশা/পদবী)

1=Doctor (ডাক্তার)

2=Nurse (নার্স/সেবিকা)

3=Ancillary worker (ওয়ার্ড পার্সন/সাহায্যকারী কর্মী)

4=Lab technician/assistant (ল্যাব টেকনিসিয়ান/এসিসট্যান্ট)

10. Date of joining in service (র্কমস্থলে যোগদানের তারিখ): …../……/………

11. Workplace (**কর্মস্থল**)

1. Administration (প্রশাসন)
2. Medical ward (মেডিকেল ওয়ার্ড)
3. ICU (আইসিইউ)
4. |Gynae andObstetric (ধাত্রীবিদ্যা বিভাগ)
5. lab (ল্যাব)

12. What is the total duration of your service as a health care worker?
(একজন স্বাস্থ্যকর্মী হিসাবে আপনার এই কর্মস্থলে যোগদানের মোট সময়কাল কতদিন?) ....

13. Household /family members (খানা/পরিবারের মোট সদস্যসংখ্যা): □□

14. Have you ever lived in the same house with someone who had TB? (যক্ষা রোগীর সাথে একই বাড়িতে কখনো থেকেছেন কি?)

1=Yes (হ্যাঁ)

2=No, (না)

88=Refused (বলতে রাজি হয়নি)

99=Unsure (নিশ্চিত না)

15. Do you have any contact with pulmonary TB patients in hospital? (আপনি কখনো হাসপাতালে কোন যক্ষা রোগীর সংস্পর্শে এসেছেন াক?)

    1=Yes (হ্যাঁ)

    2=No (না)

    99=Unsure (নিশ্চিত না)

16. Do you have any contact with pulmonary TB patients outside hospital? (আপনি কখনো হাসপাতালের বাইরে কোন যক্ষা রোগীর সংস্পর্শে এসেছেন াক?)

    1=Yes (হ্যাঁ)

    2=No (না)

    99=Unsure (নিশ্চিত না)

17. Number of years working in wards with pulmonary TB patients (পালমোনারী যক্ষারোগীর ওয়ার্ডে কতবছর ধরে কাজ করেন?)..

18. Have you ever performed or assisted in sputum collection? (আপনি কি কখনও নিজে থুতু সংগ্রহ করেছিলেন বা সাহায্য করেছিলেন)

    1=Yes (হ্যাঁ)

    2=No (না)

19. Have you ever use N95 respirators while cared for TB patients? (যক্ষারোগীর যত্ন নেয়ার সময় আপনি কি কখনও এন৯৫ মাস্ক ব্যবহার করেছিলেন?)

    1=Yes (হ্যাঁ)

    2=No (না)

20. Have you ever use mask while cared for TB patients? (যক্ষারোগীর যত্ন নেয়ার সময় আপনি কি কখনও মাস্ক ব্যবহার করেছিলেন?)

    1=Yes (হ্যাঁ)

    2=No (না)

    If yes, what type of mask? (হ্যাঁ হলে, সেটা কি ধরনের মাস্ক)..................

23. Do you have pleuritic chest pain (i.e., pain in your chest that gets worse with breathing, coughing, or moving)? [আপনার কি প্লিউরিটিক বুকে ব্যাথ্যা (pleuritic chest pain) আছে (বিশেষ দ্রষ্টব্যঃ আপনার বুকে ব্যাথা কি শ্বাস নেওয়ার সময়, কাশি/কফ বা চলাচলের সময় ব্যাপক ভাবে অনুভব হয়) ?]

1=Yes (হ্যাঁ)

2=No (না)

88=Refused (প্রত্যাখ্যান/ বলতে রাজি হয়নি)

99=Unsure (নিশ্চিত না)

24. Have you had unintended weight loss? (আপনার কি অনিচ্ছাকৃত ভাবে ওজন হ্রাস হয়েছিল?)

1=Yes (হ্যাঁ)

2=No (না)

88=Refused (প্রত্যাখ্যান/ বলতে রাজি হয়নি)

99=Don’t know (জানি না)

25. If yes, then specify quantity (kgs) যদি হ্যাঁ হয়, তবে কতটুকু (কেজি)ঃ: _________

26. Have you had fever ≥2 weeks? আপনার কি ≥ ২ সপ্তাহ ধরে জ্বর ছিল?

1=Yes (হ্যাঁ)

2=No (না)

88=Refused (প্রত্যাখ্যান/ বলতে রাজি হয়নি)

99=Don’t know (জানি না)

27. Did you have the habit of smoking? (আপনি কি ধূমপান করেন?)

☐ 1=Yes (হ্যাঁ)   ☐ 2= No (না)   ☐ 88= Refused to answer (প্রত্যাখ্যান)
