# Supplementary material for: A tuberculin skin test survey among healthcare workers in two public tertiary care hospitals in Bangladesh
Source: PLoS One. 2020 Dec 17;15(12):e0243951. doi: 10.1371/journal.pone.0243951 (PMC7745963; doi:10.1371/journal.pone.0243951)
Supplement: Attachment — Submitted filename: 2020 Nov8_Response to reviewers comments_PONE-D-20-30710.doc [file pone.0243951.s003.doc]

PONE-D-20-30710

To

Frederick Quinn
Academic Editor
PLOS ONE

Dear Frederick Quinn:

We are thankful to the reviewers for their valuable feedback. We are also grateful to the editor for allowing us to respond to the comments. Based on the helpful feedback, we revised the manuscript and believe it is more precise, clear, and informative. The following is an itemized list of our specific responses to the reviewers' comments. We have highlighted the changes in the manuscript (marked) as well.

As suggested, we have also revised the data availability statements. A minimal dataset that supports the study has been attached as a supporting document. icddr,b’s department of research administration maintains a data repository anda copy of the complete dataset will remain in the repository. Interested researchers may contact Ms. Armana Ahmed, head of research administration (aahmed@icddrb.org), for approval and full data access.

We would appreciate your further review. Please contact me directly with any additional questions or comments. We look forward to hearing from you.

Sincerely

Md. Saiful Islam

Corresponding Author

saiful@icddrb.org

***Comments:*** When submitting your revision, we need you to address these additional requirements. Please ensure that your manuscript meets PLOS ONE's style requirements, including those for file naming. The PLOS ONE style templates can be found at

https://journals.plos.org/plosone/s/file?id=wjVg/PLOSOne_formatting_sample_main_body.pdf and<https://journals.plos.org/plosone/s/file?id=ba62/PLOSOne_formatting_sample_title_authors_affiliations.pdf>

***Response:*** Thank you. The manuscript and the authors’ affiliation have been updated according to the PLOS ONE’s style requirements.

***Comments:*** 2. Regarding data availability, please note that PLOS journals require authors to make all data underlying the findings described in their manuscript fully available without restriction, with rare exception. PLOS requires that a “minimal data set” is shared, defined as the data set used to reach the conclusions drawn in the manuscript with related metadata and methods, and any additional data required to replicate the reported study findings in their entirety. Authors do not need to submit their entire data set if only a portion of the data were used in the reported study. Also, authors do not need to submit the raw data collected during an investigation if the standard in the field is to share data that have been processed. Please submit the following data: The values behind the means, standard deviations and other measures reported; The values used to build graphs; The points extracted from images for analysis.” Please review http://journals.plos.org/plosone/s/data-availability#loc-faqs-for-data-policy. If you are unable to share the data, this may result in manuscript rejection.

***Response****:* As recommended, we shared a minimal dataset.

***Comment:*** 3. Please include additional information regarding the survey or questionnaire used in the study and ensure that you have provided sufficient details that others could replicate the analyses. For instance, if you developed a questionnaire as part of this study and it is not under a copyright more restrictive than CC-BY, please include a copy, in both the original language and English, as Supporting information, or include a citation if it has been published previously.

***Response:*** As suggested, we have uploaded a copy of the questionnaire used in the survey.

***Comment:*** 4. In the Methods, please discuss whether and how the questionnaire was validated and/or pre-tested. If these did not occur, please provide the rationale for not doing so.

***Response:*** Thank you. We added, **“**The tool, adopted from a previous study, was updated and pre-tested for cognitive test among five HCWs in the study hospitals [1].” on page 6 in the manuscript (clean version).

**Comment:** 5. In your statistical analyses, please state whether you accounted for clustering by hospital. For example, did you consider using multilevel models?

***Response:*** Thanks for your comment. Based on your suggestion, we have revised the method and the analysis, and updated the table 2 and the text in the manuscript. Under the method section, we added, “In the multivariate analysis, we used a generalized linear model adjusting for hospital level clustering effect (using ‘glm.cluster’ command from the *miceadds* R package)” on page 7.

***Comments:*** 6. Please revise overstated conclusions and statements that imply causal association. For example your statement that, "...poor implementation of TB IPC in public tertiary care teaching hospitals increased the risk of LTBI among HCWs...," implies causation. A cross-sectional study cannot provide sufficient evidence for causal inferences should be avoided and instead indicate that associations were observed.

***Response:*** Based on your suggestion, we have revised the conclusion as, “This study identified high prevalence of LTBI among HCWs. This may be due to the level of exposure to pulmonary TB patients, and/or limited use of personal protective equipment along with poor implementation of TB IPC in the hospitals. Considering the high prevalence of LTBI, we recommend the national TB program consider providing preventative therapy to the HCWs as the high-risk group, and implement TB IPC in the hospitals” on page 2.

***Comments:*** 7.We note that you have indicated that data from this study are available upon request. PLOS only allows data to be available upon request if there are legal or ethical restrictions on sharing data publicly. For information on unacceptable data access restrictions, please see http://journals.plos.org/plosone/s/data-availability#loc-unacceptable-data-access-restrictions.

In your revised cover letter, please address the following prompts:

a) If there are ethical or legal restrictions on sharing a de-identified data set, please explain them in detail (e.g., data contain potentially identifying or sensitive patient information) and who has imposed them (e.g., an ethics committee). Please also provide contact information for a data access committee, ethics committee, or other institutional body to which data requests may be sent.

b) If there are no restrictions, please upload the minimal anonymized data set necessary to replicate your study findings as either Supporting Information files or to a stable, public repository and provide us with the relevant URLs, DOIs, or accession numbers. Please see http://www.bmj.com/content/340/bmj.c181.long for guidelines on how to de-identify and prepare clinical data for publication. For a list of acceptable repositories, please see http://journals.plos.org/plosone/s/data-availability#loc-recommended-repositories.

We will update your Data Availability statement on your behalf to reflect the information you provide.[Note: HTML markup is below. Please do not edit.]
***Response:*** Now, we have revised the statements. A minimal dataset that supports the study has been attached as a supporting document.

**Review Comments to the Author**

**Reviewer #1:**

***Comment:*** What about the incidence of tuberculosis in Bangladesh?

***Response:*** We have added this information, “Bangladesh shares 3.6% of the global total of 10 million people estimated with TB diseases in 2019[2]. Bangladesh is one of the 22 high TB burden countries in the world with an estimated incidence for all forms of TB in 2019 was 221 (uncertainty interval: 161-291) per 100 000 population[2] on page 4 (Manuscript clean version).

**Comment:** What about the program of BCG vaccination in Bangladesh?

***Response:*** Under the introduction, we have added this information, “In line with the country's TB epidemiology, Bangladesh's extended program on immunization has included neonatal bacillus Calmette-Guérin (BCG) vaccination nationwide since the 1980s [3]. The national coverage of the BCG vaccine was 86% in 1991; 95% in 2000; and 99% since 2013[3]” on page 4.

***Comments:*** Did you use the QFT-TB gold test for diagnosis of TB infection or TB disease in Bangladesh? If yes, why you didn't use QFT and TST tests for this study of screening of risk to TB infection?

***Response:*** Thank you for your valuable comments. QuantiFerron-Gold-in-Tube test (QFT-GIT) is expensive and is not routinely use in Bangladesh. Moreover, QFT-GIT requires a specialized laboratory for sample processing that was not widely available outside Dhaka during the time we conducted this study. Therefore, we could not use the QFT-GIT test.

***Comment:*** Did you have the information of participants about different risk factors like asthma, diabetes or others factors?

***Response:*** No, we did not collect data on asthma and diabetes. However we collected information on smoking history. Please see page 8 and the table 1 and table 2.

***Comments:*** Could you explain the choice of the references of each parameter for calculation of odd ratio for tables 2 and 3?

***Response:*** The reference category was chosen either with the highest frequency or the group with a lower risk of infection based on published literature.

**Reviewer #2:**

***Comment:*** 1. Title: a long one, no need for the question included,
a. Are healthcare workers in public tertiary care general hospitals at risk of TB infection?

***Response:*** Thank you. We have revised the title as, “A tuberculin skin test survey among healthcare workers in two public tertiary care hospitals in Bangladesh."

***Comment:*** b. all of us know the answer, so better remove it,

***Response:*** As suggested, we have removed “Are healthcare workers in public tertiary care general hospitals at risk of TB infection?” from the title.

***Comment:*** c. and only keep title starting A tuberculin skin test survey in two public tertiary care hospitals, Bangladesh

***Response:*** Thank you. We have revised the title as, ““A tuberculin skin test survey among healthcare workers in two public tertiary care hospitals in Bangladesh."

***Comment:*** d. you cannot use; Prevalence of latent tuberculosis infection among healthcare workers, Bangladesh, since its done in two specific hospitals that does not represent Bangladesh in general

Response: We revised it as, “Prevalence of latent tuberculosis infection among healthcare workers in two public tertiary care hospitals in Bangladesh”.

Comment: 2. ABSTRACT:
a. in multivariate analysis need to remove age and work years, because of problem of colinearity

***Response:*** Thank you. On page 7, we mentioned, “To check multicollinearity, we performed a variance inflation factor (VIF) analysis, and factors with VIF> 5, we considered them collinear and excluded from the multivariate model [4].

We checked for multicollinearity, and the variables with <5 variance inflation factors have been added in the multivariate analysis. Please see the outcome of the multicollinearity test.

***Comment:*** b. there should be no discussion in abstract, you mean conclusion

***Response:*** Thank you. We have revised it.

***Comment:*** c. second part of conclusion is not accepted because it is not based on study results.

***Response:*** We have revised the concluding sentence. The revised sentence is, “This study identified high prevalence of LTBI among HCWs. This may be due to the level of exposure to pulmonary TB patients, and/or limited use of personal protective equipment along with poor implementation of TB IPC in the hospitals. Considering the high prevalence of LTBI, we recommend the national TB program consider providing preventative therapy to the HCWs as the high-risk group, and implement TB IPC in the hospitals on pages 2 (manuscript clean version).

**Comment:** 3. Introduction is poor, need more elaboration on LTB as a major health problem for HCWs

Response: Thank you. We have now added more information to strengthen the introduction. On page 3 we added more information and revised it as, “In 2019, the WHO estimated that 10 million people developed TB disease globally, of whom 44% were from South-East Asia [2]. Moreover, 22,314 healthcare workers (HCWs) developed TB in the same year, with most coming from high TB burden countries in Asia [2]. Hospital HCWs in high TB burden countries are at increased risk of TB infection due to their exposure to a higher number of pulmonary TB patients than the hospital HCWs working in low TB-incidence countries [5]. A recent mathematical modelling study on the global burden of latent TB infection (LTBI) estimated that around 1.7 billion people are infected with LTBI in the world [6]. In a systematic review of 18 studies from seven high TB burden countries, the prevalence of LTBI among HCWs was reported to be 47% (95% CI 34- 60)[7]. This risk may be high among HCWs who work in health facilities that lack proper infrastructure and limited implementation of TB infection prevention and control (IPC) healthcare measures. The risk of LTBI among different health care workers may vary by place of work, duration of exposures, and compliance with TB IPC measures [8]. Prior studies identified considerable heterogeneity in the risk of LTBI among different occupations and reported high risk among doctors, nurses, and ancillary workers [8, 9]. HCWs that were most likely to be infected had the most prolonged duration and extent of patient contact [9, 10].

People with LTBI represent a reservoir for potential TB disease [6]. Overall, without treatment, about 5 to 10% of infected persons may develop TB disease at some time in their lives, and the active stage frequently occurs within the first two years after infection [11, 12]. People with an impaired immune system are at increased risk of developing TB disease than persons with standard immune systems[12]. Diabetes and smoking behavior also increase TB disease risk among the person with LTBI[13].

On page 4, we added, “Bangladesh shares 3.6% of the global total of 10 million people estimated with TB diseases in 2019[2]. Bangladesh is one of the 22 high TB burden countries in the world with an estimated incidence for all forms of TB in 2019 was 221 (uncertainty interval: 161-291) per 100 000 population[2]. In line with the country's TB epidemiology, Bangladesh's extended program on immunization has included neonatal bacillus Calmette-Guérin (BCG) vaccination nationwide since the 1980s [3]. The national coverage of the BCG vaccine was 86% in 1991; 95% in 2000; and 99% since 2013[3].”

***Comment:*** 4. Review of literature missing key and important publications in the context

***Response:*** Thank you. I would like to request you to see our response under the previous comment. We have updated literature review and added recent publications.

***Comment:*** 5. In results 1st paragraph, 169 refused to participate, why, need explanation on that.

***Response:*** On page 10, we added, “One hundred and fifty nine HCWs did not consent for TST due to their illness, prior history of allergic reaction to TST, unavailability for TST reading, and past history of active TB disease”.
***Comments:*** 6. The last part of results are comments that have no tables, where are tables for use of masks and exposure times.

***Response:*** We have now added the information in table 2.

| **Demography and exposures** | **TST positive % (n/N)** | | **TST negative % (n/N)** | **OR*(95% CI*)** | **aOR*(95% CI*)** |
| --- | --- | --- | --- | --- | --- |
| **Hours working per day** | | | | | |
| <6 hours | | 43 (15/35) | 57(20/35) | **Reference** |  |
| 6 hours and above | | 43(303/704) | 57 (401/704) | **1.01 (0.51-2.03)** |  |
| **Use of mask or respirator** | | | | | |
| Yes | 40 (75/190) | | 60 (115/190) | 0.85(0.61-1.19) |  |
| No | 43 (271/626) | | 57 (355/626) | Reference |  |

***Comments:*** 7. Where are the conclusion and recommendation?

***Response:*** On Page 13 and 14, we described the conclusion and recommendations, " In conclusion, this study identified a high number of HCWs was infected with LTBI in the study facilities. This study represents a first step in estimating the risk of TB exposures and LTBI among HCWs in public tertiary care hospitals in Bangladesh. Our study identified the importance of preventing or interrupting the occupational risk of TB among HCWs. Considering the high prevalence of LTBI among the HCWs, we recommend that NTP consider providing preventative therapy to the HCWs as the high-risk group. Our analysis also warrants the immediate implementation of TB IPC in Bangladeshi tertiary care teaching hospitals. Our study also recommends relocation or renovation of the nursing workstation to minimize exposure for staff. Separating the nursing station with transparent curtains or glass could be an option. We recommend establishing a TB IPC program in all tertiary care teaching hospitals to ensure proper implementation of the TB IPC healthcare measures. Finally, an assessment of the HCWs private chambers and private clinics should be done to recommend context-appropriate modifiable TB IPC measures, including improving the facilities' physical layout. The study findings will assist Bangladesh’s national TB control program to effectively revise the TB IPC policy and reduce healthcare-associated TB infection.”

***References:***
